# Soluble Epoxide Hydrolase Inhibitory Activity of Components Isolated from *Apios americana* Medik

**DOI:** 10.3390/molecules22091432

**Published:** 2017-08-30

**Authors:** Jang Hoon Kim, Hyo Young Kim, Si Yong Kang, Young Ho Kim, Chang Hyun Jin

**Affiliations:** 1 Advanced Radiation Technology Institute, Korea Atomic Energy Research Institute, Jeoungeup, Jeollabuk-do 56212, Korea; oasis5325@gmail.com (J.H.K.); khy5012@kaeri.re.kr (H.Y.K.); sykang@kaeri.re.kr (S.Y.K.); 2 College of Pharmacy, Chungnam National University, Daejeon 34134, Korea; yhk@cnu.ac.kr

**Keywords:** *Apios americana*, leguminosae, sEH inhibitor, molecular simulation

## Abstract

A new compound **1**, 5-methoxy-2,5,7,4′-tetrahydroxy-coumaronochromone, along with seven known compounds (**2**–**8**), were isolated from *Apios americana* using open column chromatography. Their structures were established based on an analysis of 1D and 2D NMR, and MS spectra. Among these, two compounds **1** and **2** showed inhibitory activity on soluble epoxide hydrolase (sEH) at a concentration below 50 μM. The respective competitive (**1**) and mixed (**2**) inhibitors were revealed to have *K*_i_ values of 21.0 ± 0.8 and 14.5 ± 1.5 μM, based on the Dixon plot. The potential inhibitor (**2**) was visually presented in a predicted binding pose in the receptor by molecular docking. Additionally, molecular dynamics were performed for a detailed understanding of their complex by Gromacs 4.6.5 package.

## 1. Introduction

Groundnut (*Apios americana* Medik) is a perennial herb of the Leguminosae family, and is native to the temperate and eastern regions of North America [1]. The tubers of *A. americana* grow to a depth of about 100 cm, and form knots in intervals of about 5 and 10 cm [1,2]. Groundnuts have been used as a traditional health food by the North American Indians [2,3]. The nutrients of these roots were previously reported to include fatty acid, amino acid, and carbohydrate compositions, and a high content of protein (over 12%) [3,4]. Recently, isoflavonoids, such as genistein, barpisoflavone A, 2′-hydroxygenistein, 5-methylgenistein, 2′-hydroxy genistein-7-*O*-gentibioside, 2′-hydroxy,5-methoxy genistein-4′,7-*O*-diglucoside, and 2′-hydroxy genistein-7-*O*-glucoside, were isolated from the tubers of *A. americana* [3,5]. Of these, genistein and 2′-hydroxygenistein were revealed to show binding activities with estrogen receptors. Isoflavonoid glycosides, 2′-hydroxy genistein-7-*O*-gentibioside and 2′-hydroxy genistein-7-*O*-glucoside blocked the binding of [^3^H]-dihydrotestosterone with androgen receptors [3,5].

Soluble Epoxide Hydrolase (EC 3.3.2.10, sEH), one of the α/β hydrolase enzymes with a C-terminal domain of about 35 kDa, hydrolyzes the epoxide of four major isomers of eicosatrienoic acids (EETs), namely 5,6-EET, 8,9-EET, 11,12-EET, and 14,15-EET, from arachidonic acid [6,7]. The active site of sEH contains a catalytic triad composed of Asp333, Asp495, and His523 [8]. The Asp333 residue acts as a nucleophile leading to the bridge of the α-hydroxyl acyl-enzyme complex, and the Asp495/His523 residues with a water molecule participate in the conversion of the complex to the corresponding dihydrodiol [9,10]. In particular, the mutation of His523 was revealed to dramatically decrease the catalytic reaction of sEH [10]. It was proven that this residue operated as the key amino acid in the catalytic mechanism. EETs were known to have a variety of biological properties which are anti-inflammatory, vasodilatory, and cardiovascular. Additionally, they suppressed NF-κB activation and VCAM-1 expression [6,11,12]. Recently, urea-type compounds, 12-(3-adamantan-1-yl-ureido)dodecanoic acid (AUDA), *trans*-4-[4-(3-adamantan-1-yl-ureido)-cyclohexyloxyl]-benzoic acid (*t*-AUCB), and 1-trifluoromethoxy phenyl-3-(1-acetylpiperdin-4-yl)urea (TPAU), were developed as potential inhibitors to block the catalytic reaction of sEH for the treatment of cardiovascular disease [6,11]. However, their use was limited due to poor solubility and rapid metabolism [11]. To overcome these problems, the scientists researched to find new inhibitors form natural plants. They reported that biflavonoid, selaginellin, stilbene, and anthraquinone derivatives had inhibitory activity on sEH [6,7,8,11].

The objective of our study is to isolate and identify isoflavonoids from *A. americana*, to evaluate the inhibitory activity of new-type compounds on sEH in vitro, and to provide the information of the interaction between receptor and ligand.

## 2. Results and Discussion

### 2.1. Isolation and Identification

**Figure 1:**
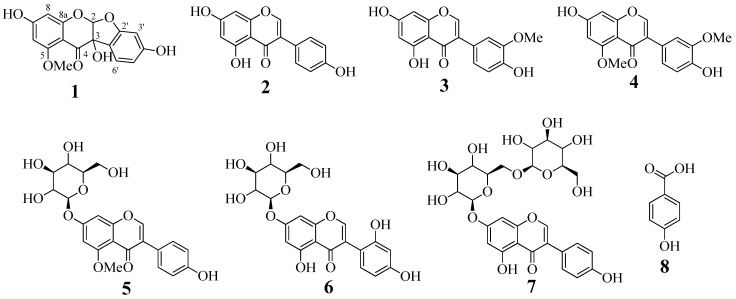
The structure of isolated compounds **1**–**8** from *A. americana*.

The methanol extract of the roots of *A. americana* was progressively fractionated into *n*-hexane, chloroform, ethyl acetate, butanol, and water. Ethyl acetate fraction was subjected to open column chromatographies over silica gel, C-18, and sephadex LH-20, to obtain a new isoflavonoid, 5-methoxy-2,5,7,4′-tetrahydroxy-coumaronochromone (**1**), along with genestein (**2**) [5,13], 3′-methoxy-4′,5,7-trihydroxyisoflavone (**3**) [13], gerontoisoflavone A (**4**) [5], 5-methoxygenistein-7-*O*-glucoside (**5**) [3], 2′-hydroxygenistein-7-*O*-glucoside (**6**) [3], 2′-hydroxygenistein-7-*O*-gentibioside (**7**) [3], and 4-hydroxybenzoic acid (**8**) [14] (Figure 1). The structures were elucidated by a comparison of 1D- and 2D-NMR, and HR-ESIMS data, with those reported previously.

**Figure 2:**
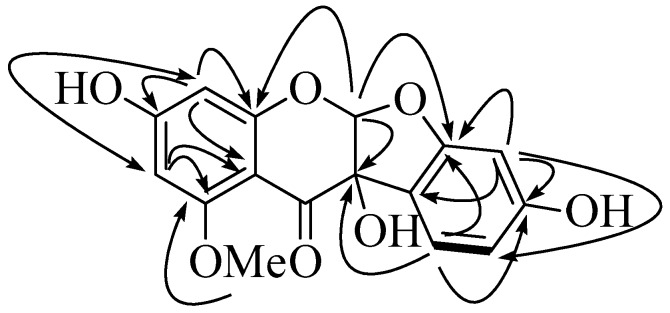
Correlation between key COSY (—) and HMBC (→) of compound **1.**

Compound **1** was obtained as a white powder with $[\alpha {]}_{D}^{20}$ +44.0° (*c* 0.1, MeOH). The molecular formula of C_16_H_12_O_7_ was determined by HR-ESI-Ms (339.0502 *m*/*z* [M + Na]^+^, calcd. for 339.0475). The ^1^H-NMR spectrum of **1** indicated the presence of five aromatic (*δ*_H_ 7.08 (1H, d, *J* = 8.4 Hz), 6.36 (1H, dd, *J* = 8.4, 1.5 Hz), 6.28 (1H, d, *J* = 1.5 Hz), 6.11 (1H, d, *J* = 2.2 Hz), 6.03 (1H, d, *J* = 2.2 Hz)), a methine (*δ*_H_ 6.14 (1H, *s*)), and a methoxy (*δ*_H_ 3.76 (3H, *s*)) signals. The ^13^C-NMR spectrum displayed the presence of sixteen carbon signals, containing one ketone (*δ*_C_ 189.0), twelve aromatic (*δ*_C_ 167.3, 163.8, 163.0, 162.4, 161.9, 126.5, 119.2, 110.6, 104.9, 98.7, 98.4, 95.8), a methine (*δ*_C_ 122.5), a quaternary carbon (*δ*_C_ 82.2), and a methoxy group (*δ*_C_ 56.8). The ^1^H-^1^H COSY spectrum displayed a correlation between H-5′ [*δ*_H_ 6.36 (1H, dd, *J* = 8.4, 1.5 Hz)] and H-6′ (*δ*_H_ 7.08 (1H, d, *J* = 8.4 Hz)). The HMBC spectrum showed correlations from H-6′ (*δ*_H_ 7.08 (1H, d, *J* = 8.4 Hz)) to C-3 (*δ*_C_ 82.2), C-2′ (*δ*_C_ 163.0), and C-4′ (*δ*_C_ 162.4); from H-3′ (*δ*_H_ 6.28(1H, d, *J* = 1.5 Hz)) to C-1′ (*δ*_C_ 119.2), C-4′ (*δ*_C_ 162.4), and C-2′ (*δ*_C_ 163.0); from H-2 (*δ*_H_ 6.14 (1H, s)) to C-3 (*δ*_C_ 82.2), C-8a (*δ*_C_ 161.9), and C-2′ (*δ*_C_ 163.0); from H-8 (*δ*_H_ 6.11 (1H, d, *J* = 2.2 Hz)) to C-6 (*δ*_C_ 98.4), C-7 (*δ*_C_ 167.3), C-8a (*δ*_C_ 161.9), and C-4a (*δ*_C_ 104.9); from H-6 (*δ*_H_ 6.03 (1H, d, *J* = 2.2 Hz)) to C-8 (*δ*_C_ 95.8), C-4a (*δ*_C_ 104.9), and C-5 (*δ*_C_ 163.8). The correlation of the methoxy proton signal (*δ*_H_ 3.76) with C-5 (*δ*_C_ 163.8) confirmed that the methoxy group was connected to the oxygen atom at the C-5 position (Figure 2, Table 1 and Figures S1–S5). Additionally, the ^1^H- and ^13^C-NMR data were compared with previous reports [15]. The structure of compound **1** was identified and named 5-methoxy-2,5,7,4′-tetrahydroxy-coumaronochromone.

### 2.2. Enzyme Activity

**Figure 3:**
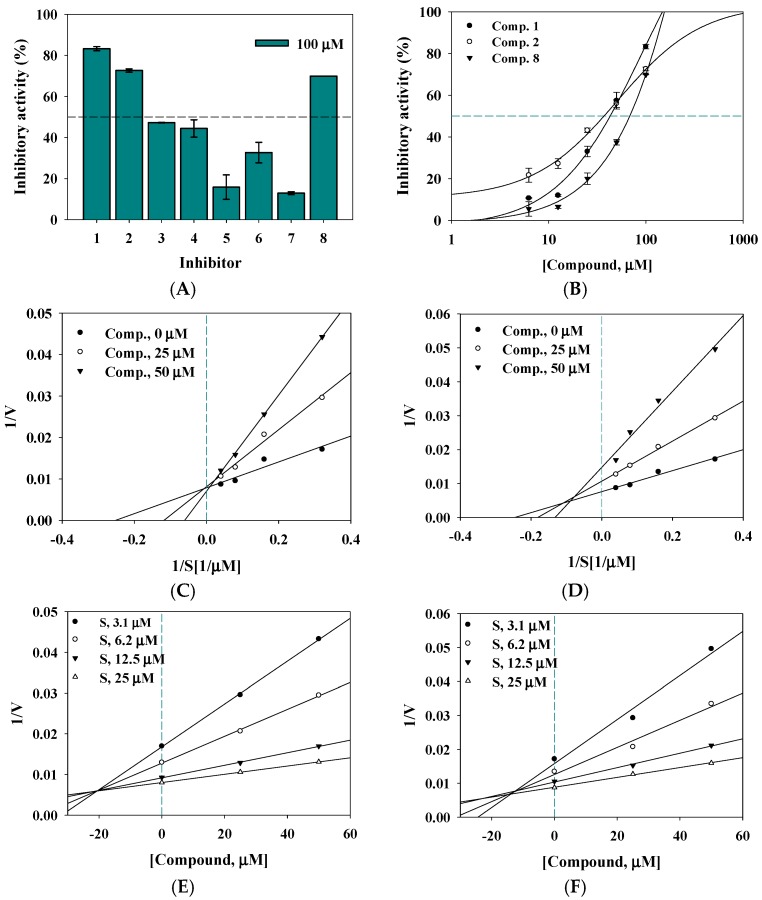
Inhibitory activity on sEH, of compounds extracted from *A. americana*: compounds **1**–**8** (**A**); and compounds **1**, **2**, and **8** (**B**); Lineweaver–Burk plot and Dixon plot, respectively: compound **1** (**C**,**E**) and compound **2** (**D**,**F**).

To evaluate the inhibitory activity of isolated compounds **1**–**8** from *A. americana* on sEH, the inhibition of 6-methoxynaphtaldehyde from 3-phenyl-cyano(6-methoxy-2-naphthalenyl) methyl ester-2-oxiraneacetic acid was evaluated, using fluorometeric determination with a TECAN infinite F200 Pro microplate reader (Tecan Trading AG, Männedorf, Switzerland). AUDA was used as the positive control (IC_50_ value = 7.6 ± 2.5 nM). To find the potential inhibitor of sEH among these fractions, all the isolated compounds **1**–**8** were tested at 100 μM in vitro (Figure 3A). All exhibited inhibitory ratios, ranging from 12.8 ± 0.7 to 83.2 ± 1.0% of the control value. Compounds **1**, **2**, and **8** showed inhibitory rates over 50%, and were further evaluated at concentrations ranging from 6.2 to 100 μM, to elucidate the IC_50_ values. These inhibitors (**1**, **2**, and **8**) showed dose-dependent inhibition, with IC_50_ values of 43.2 ± 0.4, 33.5 ± 0.8, and 69.3 ± 1.9 μM, respectively (Figure 3B and Table 2).

### 2.3. Enzyme Kinetics

To determine the nature of the binding mechanism between the inhibitors (**1** and **2**) and the enzyme, two inhibitors were diluted as concentrations of 0, 25, and 50 μM in methanol, and the respective samples were then evaluated for their inhibitory activity at various substrate concentrations ranging from 3.1 to 25 μM. The enzyme kinetics of compounds **1** and **2** were determined by generating double-reciprocal plots (Lineweaver–Burk plot and Dixon plot). As shown in Figure 3C,D, Lineweaver–Burk plots of **1** yielded a family of straight lines with different slopes through a common point on the Y-axis; therefore, **1** was designated as a competitive inhibitor with preferential binding toward activity site of receptor. On the other hand, a family of straight lines of **2** cross over one point in the second quadrant, and hence **2** was confirmed to interact with the receptor as a mixed inhibitor. Additionally, the inhibition constant (*K*_i_) values of isoflavonoids **1** and **2** were calculated by using Dixon plots (Figure 3E,F). The *K*_i_ values for the inhibition of sEH were 21.0 ± 0.8 and 14.5 ± 1.5 μM, respectively.

### 2.4. Molecular Docking

**Figure 4:**
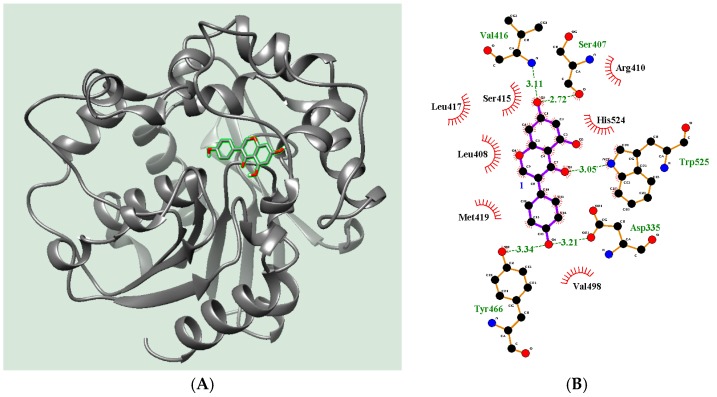
Docking pose of 3D-(**A**) and 2D-(**B**) structures of compound **2** into the predicted binding site toward sEH.

The study for visualizing the complex between receptor (code ID: 3ANS) and ligand (**2**) was subjected to molecular simulation using the Autodock 4.2 program. According to the result of enzyme kinetics, a grid was set up to contain the full receptor for scholarly molecular docking. The result was represented graphically by LIGPLOT v.4.5.4 and Chimera 1.10rc. As indicated in Figure 4A and Table 3, ligand (**2**) was docked to form a stable pose, with −8.02 kcal/mol of Autodock score, into the right pocket of the receptor, and was surrounded around twelve amino acids (Tyr466, Val498, Asp335, Trp525, His524, Arg410, Ser407, Val416, Ser415, Leu417, Leu408, and Met419). Among these, Asp335, Ser407, Val416, Tyr466, and Trp525 were hydrogen bonds at distances of 3.21, 2.72, 3.11, 3.34, and 3.05 Å from the inhibitor (**2**) (Figure 4B). Molecular docking helped keep track of the allosteric site where compound **2** may be combined. Our result showed that the complex calculated with the lowest Autodock score was the best pose, and the potential inhibitor (**2**) was found to make five hydrogen bonds with Asp335, Ser407, Val416, Tyr466, and Trp525 in the right pocket next to the active site. This study showed similar results with previous reports. The right pocket proved to be the position of sEH preferring the interaction with compounds, such as flavonoids, stilbene, and anthraquinone derivatives again [8,16].

### 2.5. Molecular Simulation

**Figure 5:**
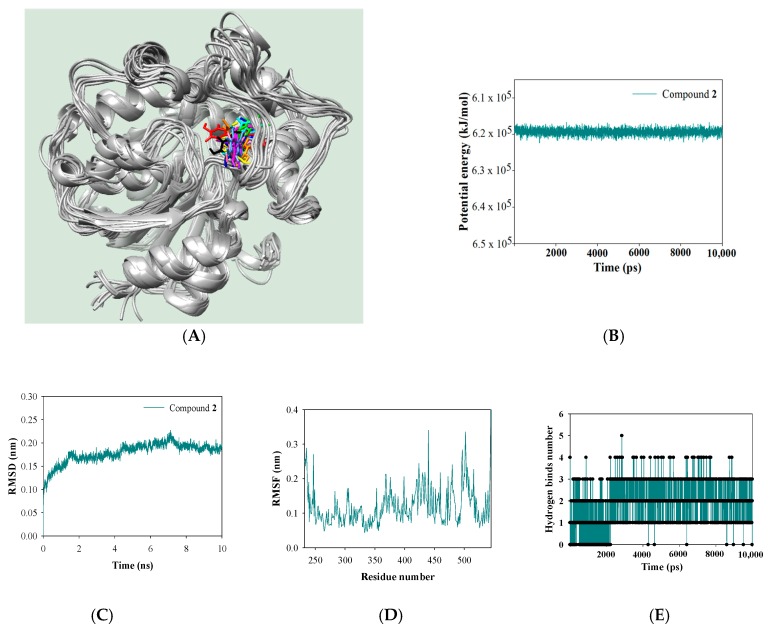

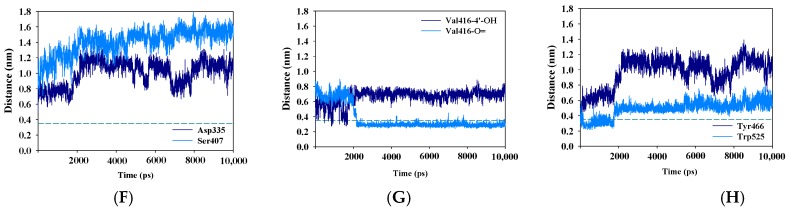
The superpositions (**A**) of the complex of receptor and ligand for the simulation time in ns (red: 0, orange: 1, yellow: 2, green: 3, cyanin: 4, blue: 5, cornflower blue: 6, purple: 7, hot pink: 8, magenta: 9, black: 10). The potential energy (**B**); RMSD (**C**); RMSF (**D**), the number of hydrogen bonds (**E**) between receptor and ligand. The interaction distance of key amino acids (**F**–**H**) with ligand (**2**).

Molecular dynamics simulation was performed to investigate the corresponding interaction of the receptor with the ligand, using the Gromacs 4.6.5 package. The key results are presented in Figure 5. The complexes were superposed at snap shot in 1 ns intervals during a 10 ns simulation cycle. Figure 5A shows the corresponding flexibility according to the loop movement at allosteric site of the receptor. This simulation was made and the final value of stable potential energy was about −6.2 × 10^5^ kJ/mol, with RMSD under 0.25 nm, and RMSF of 0.32 nm (Figure 5B–D). These results proved that molecular dynamics of the complex was perfectly achieved. Additionally, this study observed the hydrogen bonds to have important roles at the interaction of the receptor and the ligand. During the simulation, they were mainly built of 1–3 hydrogen bonds, and sometimes formed 0, 4, and 5 hydrogen bonds (Figure 5E). Finally, the distances of the key amino acids in molecular docking with ligand (**2**) were calculated during the 10 ns trajectory. As shown in Figure 5F,H, Asp335, Ser407, and Tyr466 were far away, at a distance of over 4.5 Å from the ligand (**2**) in the course of the energy minimization. Also, during molecular simulation, they were separated from the ligand at over 10 Å distance. Conversely, Val416 and Trp525 maintained a distance of 3.5 Å up to 1.8 ns into molecular dynamics. Interestingly, after 2 ns, Val416 was maintained within 3.5 Å distance of the ketone of the ligand (Figure 5G). Molecular dynamics revealed that the potential inhibitor (**2**) maintained a constant 3.5 Å distance fromVal416 of the receptor during the 10 ns simulation, and with Trp525 at about 2 ns. Resvertol, desoxyrhapontigenin, and rhapontigenin of stilbene are composed of hydrogen bonds with the Val416 residue in the right pocket [8]. Our findings were analyzed with compound **2** dependently moved according to the mobility of the loop containing Val416. These results suggest that Val416 acts as the key amino acid while **2** is interacting with the allosteric site of the receptor. Finally, compound **2** isolated from *A. americana* was observed to block the catalytic reaction of sEH, which could be targeted for the treatment of cardiovascular disease. Two hydroxyl and ketone groups of compound **2** may be closely related to Val416 of the receptor.

## 3. Materials and Methods

### 3.1. General Experimental Procedures

High-resolution electrospray ionization mass spectrometry (HR-ESIMS) were recorded on a MicroQ-TOF-III mass spectrometer (Bruker Daltonics, Bremen, Germany). Nuclear magnetic resonance (NMR) experiments were conducted on an ECA500 (JEOL, Tokyo, Japan). Thin layer chromatography analysis was performed on Kieselgel 60 F_254_ (Merck, Kenilworth, NJ, USA) plates (silica gel, 0.25 mm layer thickness); pure compounds were visualized by dipping plates into 10% (*v*/*v*) H_2_SO_4_ reagent (Sigma-Aldrich, St. Louis, MO, USA), after which they were heat treated at 300 °C for 30 s. Normal-phase silica gel (Merck 60A, 70–230 or 230–400 mesh ASTM), sephadex LH-20 (GE Healthcare Bio-Sciences, Pittsburgh, PA, USA), and reversed-phase silica gel (YMC Co., Kyoto, Japan, ODS-A 12 nm S-150, S-75 μm) resins were used for open column chromatography. AUDA (10007927), sEH (10011669), and PHOME (10009134) were purchased from Cayman (Cayman, Ann Arbor, MI, USA).

### 3.2. Plant Material

In October 2016, the roots of *A. americana* (voucher specimen RBRC 001) were cultivated and collected locally from Jeoungeup, Jeollabuk-do, Korea. The species was identified by Dr. S.Y. Kang of Radiation Breeding Research Center, Korea Atomic Energy Research Institute. 

### 3.3. Extraction and Isolation

The roots of *A. americana* (4 kg) were extracted twice with 80% methanol (80 L) at room temperature for 7 days. The combined solution was evaporated under reduced pressure to give a MeOH extract (337 g). This extract was suspended in 3 L H_2_O, and successively extracted with *n*-hexane (30 g), ethyl acetate (9 g), butanol (67 g), and aqueous (227 g) layers. The ethyl acetate fraction was subjected to silica gel column chromatography, using a chloroform-methanol gradient system (from 10:1→1:100) to yield 12 fractions (E1–E12). The E5 fraction was subjected to C-18 column chromatography by using 30–80% methanol gradient system, to obtain compounds **2** (10 mg) and **5** fractions (E51–E55). The E53 fraction was separated further with Sephadex LH-20 column chromatography using methanol, to obtain compounds **3** (13 mg) and **4** (9 mg). Compound **1** (17 mg) was purified by Sephadex LH-20 column chromatography from the E55 fraction. The E7 Fraction was separated with C-18 column chromatography using 20–60% methanol gradient system, to give compound **7** (5 mg) and six fractions (E71–E76). The compounds **5** (9 mg), **8** (5 mg), and a fraction (E741) were purified by Sephadex LH-20 column chromatography by using methanol from the E74 fraction. E741 was fractionated with C-18 column chromatography using 50% methanol, to obtain compound **6** (19 mg).

### 3.4. sEH Assay

The sEH assay was performed as described previously [8]. Briefly, 130 µL of sEH in 25.0 mM Bis-Tris-HCl buffer (pH 7.0) and 20.0 µL of the compounds (1–0.06 mM concentration) diluted in MeOH, were added in 96-well plate, to which 50.0 µL of 20.0 µM PHOME was added in the mixture. After initiating the enzyme reaction at 37 °C, the products by hydrolysis of the substrate were monitored at excitation and emission of 330 and 465 nm for one hour.

Inhibitory activity (%) = 100 − [(*C*_40_ − *C*_0_) − (*S*_40_ − *S*_0_)/(*C*_40_ − *C*_0_)] × 100 (1)

where *C*_40_ and *S*_40_ were the fluorescence of the control and inhibitor, respectively, after 40 min, *S*_0_ and *C*_0_ is the fluorescence of inhibitor and control, respectively, at 0 min. AUDA was used as the positive control.

### 3.5. Molecular Docking

The molecular docking simulation was performed as previously described [17]. The 3D structure of the ligand was built and minimized by MM2 using Chem 3D Pro. (Ver. 14.0). The flexible bonds of the ligand were assigned with AutoDockTools. The 3D structure of sEH (PDB ID: 3ANS) was obtained from RCSB (protein data bank) after substrates were removed from the original enzyme. All hydrogen atoms and gasteiger charges were assigned using AutoDockTools. Briefly, compound **2** was subjected to the simulation of the grid containing full enzyme (X: 126, Y: 126, and Z: 126) at 0.375 Å. This docking study was simulated using the Lamarckian Genetic Algorithm with Runs 50 and the long maximum number.

### 3.6. Molecular Dynamics

Molecular dynamics simulation was performed as previously reported, with few modifications [18]. The topology files of the ligand and receptor were built as GROMOS 53A6 force-field by prodrg and Gromacs 4.6.5, respectively. The gro complex of them was placed in the center of a cubic box (7.8 7.8 7.8) solvated with water molecules containing six Cl^−^ (1.0 Å distance). Energy minimization was performed to end when the minimization reached with the maximum force under 10.0 kJ/mol. Following this, the Particle Mesh Ewald (PME) method was used for the treatment of long-range electrostatic interactions, and the linear constraint solver (LINCS) algorithm was used for covalent bond constraints. Each NVT and NPT was performed for 0.1 ns to equilibrate the system with constant volume, temperature (300 K), and pressure (1 bar). The MD ran for 10 ns (10,000 ps).

### 3.7. Statistical Analysis

All assays in the presence of inhibitors were performed in triplicate. The results are presented as the means ± standard error of the mean. Sigma Plot (SPP Inc., Chicago, IL, USA) was used for analysis of results.

## 4. Conclusions

Using column chromatography, a new compound, 5-methoxy-2,5,7,4′-tetrahydroxy-coumaronochromone (**1**); along with genestein (**2**); 3′-methoxy-4′,5,7-trihydroxyisoflavone (**3**); gerontoisoflavone A (**4**); 5-methoxygenistein-7-*O*-glucoside (**5**); 2′-hydroxygenistein-7-*O*-glucoside (**6**); 2′-hydroxygenistein-7-*O*-gentibioside (**7**); and 4-hydroxybenzoic acid (**8**); were purified from *A. americana*. This study was undertaken to evaluate their inhibitory activity on sEH. Of these, compounds **1** and **2** had dose-dependent IC_50_ values of less than 50 μM, and acted as competitive and mixed inhibitors, respectively. Based on the Dixon plot, the *K*_i_ value of compound **2** was calculated at about 10 µM. Flavonoids, stilbene, and anthraquinone derivatives from medicinal plants have been revealed to be potential inhibitors of sEH [8,16]. Through this study, components from *A. americana*, which has been used as food source, were proved to be new inhibitors of the sEH enzyme which is targeted for treatment of cardiovascular disease. As indicated in Figure 3A, these aglycon derivatives were observed to have more inhibitory activity on sEH than that of glycosides. Furthermore, our study suggests for the first time the predicted pose of isoflavonoid binding to sEH by using in silico skills. The mixed-type inhibitor (**2**) was suitably fitted in the right pocket next to the sEH active site. This interaction was constantly held via the interaction of the inhibitor with residue Val416 of the enzyme. These results suggest that it is necessary to consider the hydrophobic interaction with Val416 when developing synthetic compounds based on the pharmacophore of the isoflavonoid type. Additionally, this right pocket as an allosteric site needs to be considered for the development of non-competitive or mixed-type inhibitors of sEH. 

Finally, for development of a new type inhibitor of sEH, as an alternative to urea, this study suggests that the structure of compound **2** is eligible to be the backbone of the lead compound.

## Figures and Tables

**Table 1:** ^1^H- and ^13^C-NMR data for compound **1**.

Position	*δ*_H_^a,b^	*δ*_C_^a,c^
2	6.14 (1H, s)	122.5
3		82.2
4		189.0
4a		104.9
5		163.8
6	6.03 (1H, d, *J* = 2.2 Hz)	98.4
7		167.3
8	6.11 (1H, d, *J* = 2.2 Hz)	95.8
8a		161.9
1′		119.2
2′		163.0
3′	6.28 (1H, d, *J* = 1.5 Hz)	98.7
4′		162.4
5′	6.36 (1H, dd, *J* = 8.4, 1.5 Hz)	110.6
6′	7.08 (1H, d, *J* = 8.4 Hz)	126.5
5-OMe	3.76 (3H, s)	56.8

^a^ Measured in CD_3_OD; ^b^ 500 MHz; ^c^ 125 MHz.

**Table 2:** The sEH inhibitory activities of compounds from *A. Americana*.

The Inhibitory Activity on sEH		
	IC_50_ (μM) ^a^	Binding mode (*K*_i_, μM) ^a^
**1**	43.2 ± 0.4	Competitive (21.0 ± 0.8)
**2**	33.5 ± 0.8	Mixed (12.4 ± 1.5)
**8**	69.3 ± 1.9	N.T ^c^
AUDA ^b^	7.6 ± 2.5 nM	

^a^ All compounds were tested in a set of triplicated experiment; ^b^ Positive control; ^c^ Not Tested.

**Table 3:** Interaction and Autodock score between sEH and inhibitors.

	Hydrogen Bonds (Å)	Binding Energy (kcal/mol)
**2**	Asp335 (3.21), Ser407 (2.72), Val416 (3.11), Tyr466 (3.34), Trp525 (3.05)	−8.02

## Supplementary Materials

Supplementary materials are available online.
