# Supplementary material for: Soluble Epoxide Hydrolase Inhibitory Activity of Components Isolated from Apios americana Medik
Source: Molecules. 2017 Aug 30;22(9):1432. doi: 10.3390/molecules22091432 (PMC6151598; doi:10.3390/molecules22091432)
Supplement: Supplementary file 1 [file molecules-22-01432-s001.pdf]

# Soluble Epoxide Hydrolase Inhibitory Activity of Components Isolated from *Apios americana* Medik

Jang Hoon Kim [1], Hyo Young Kim [1], Si Yong Kang [1], Young Ho Kim [2] and Chang Hyun Jin [1,*]

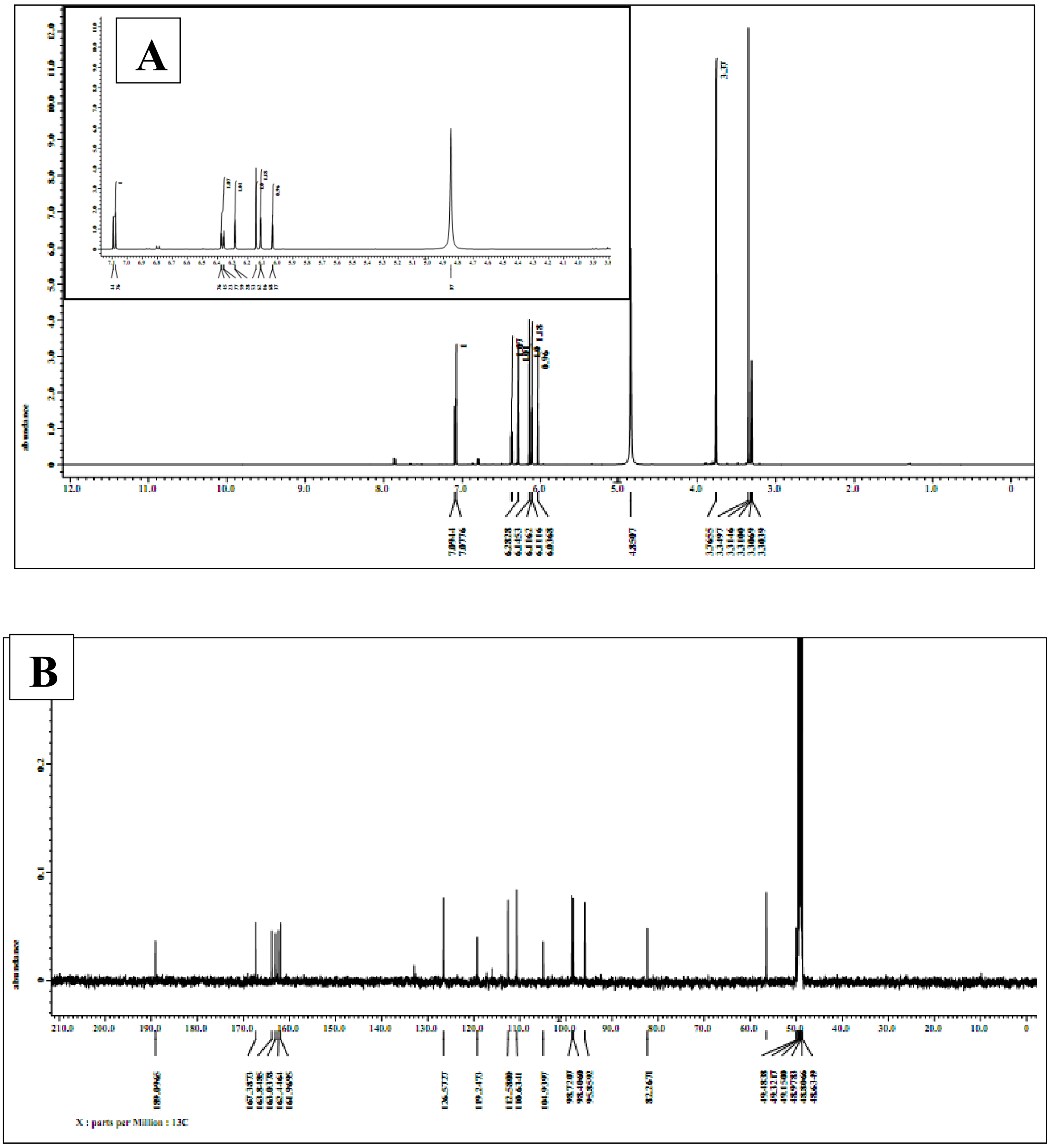


**Figure S1**. $^{1}$H(A) and $^{13}$C(B)-NMR of compound **1**.

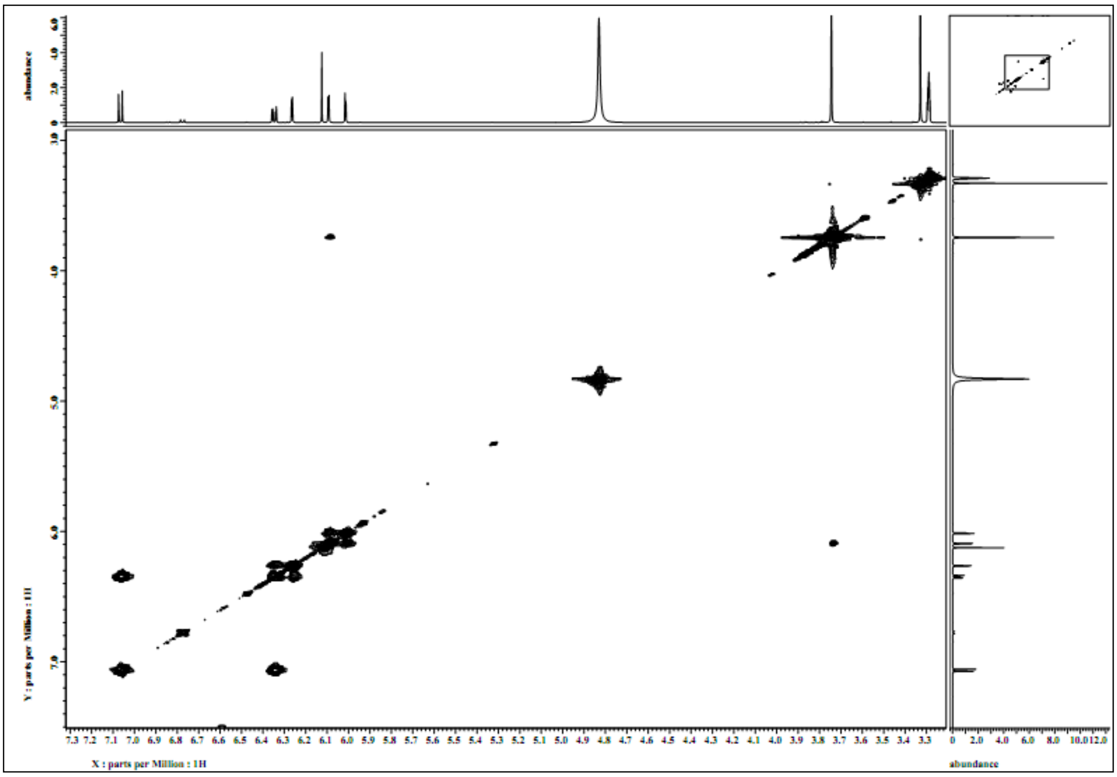

**Figure S2**. COSY of compound **1**.

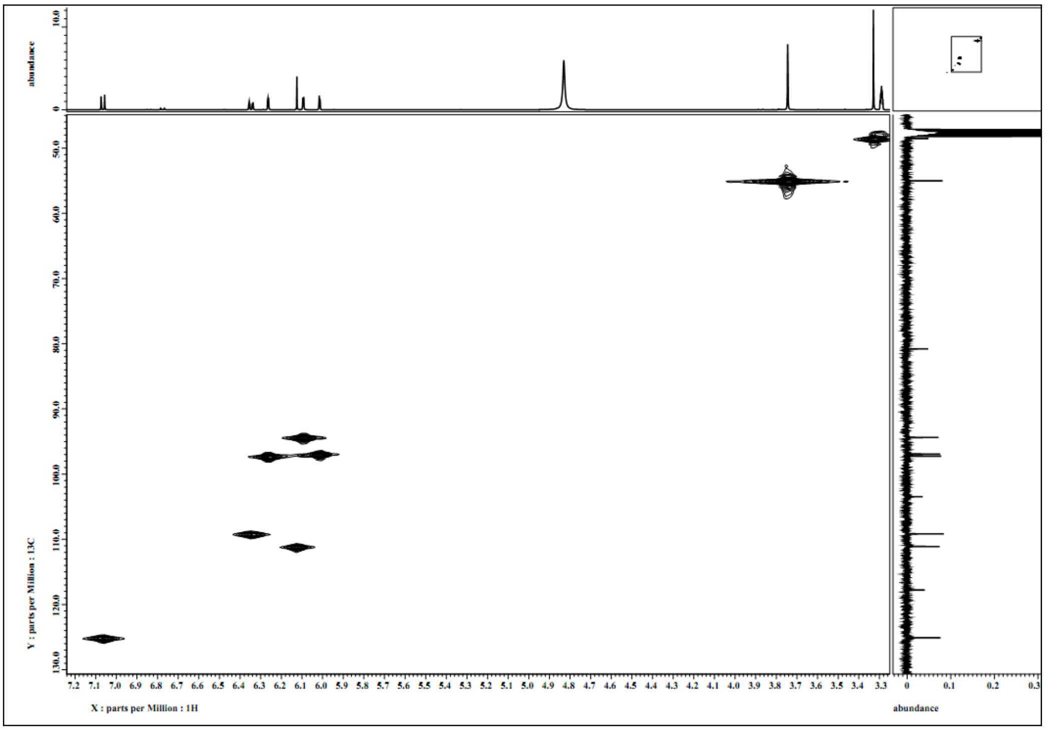

**Figure S3**. HMQC of compound **1**.

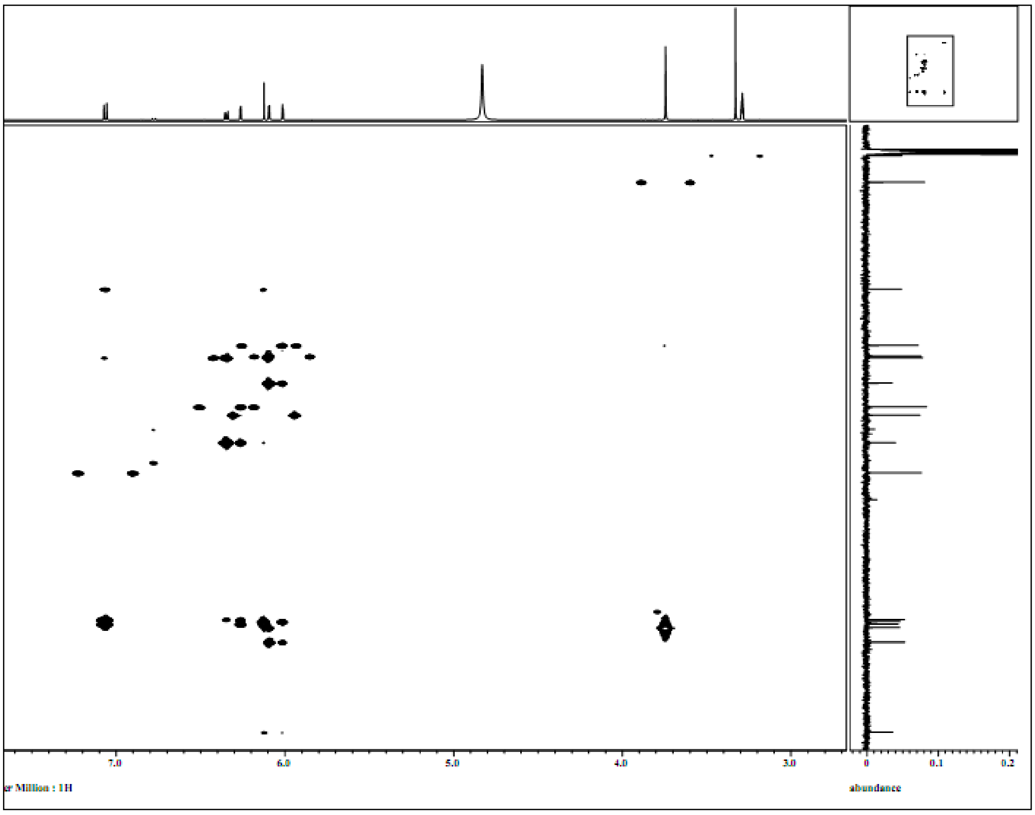


**Figure S4**. HMBC of compound **1**.

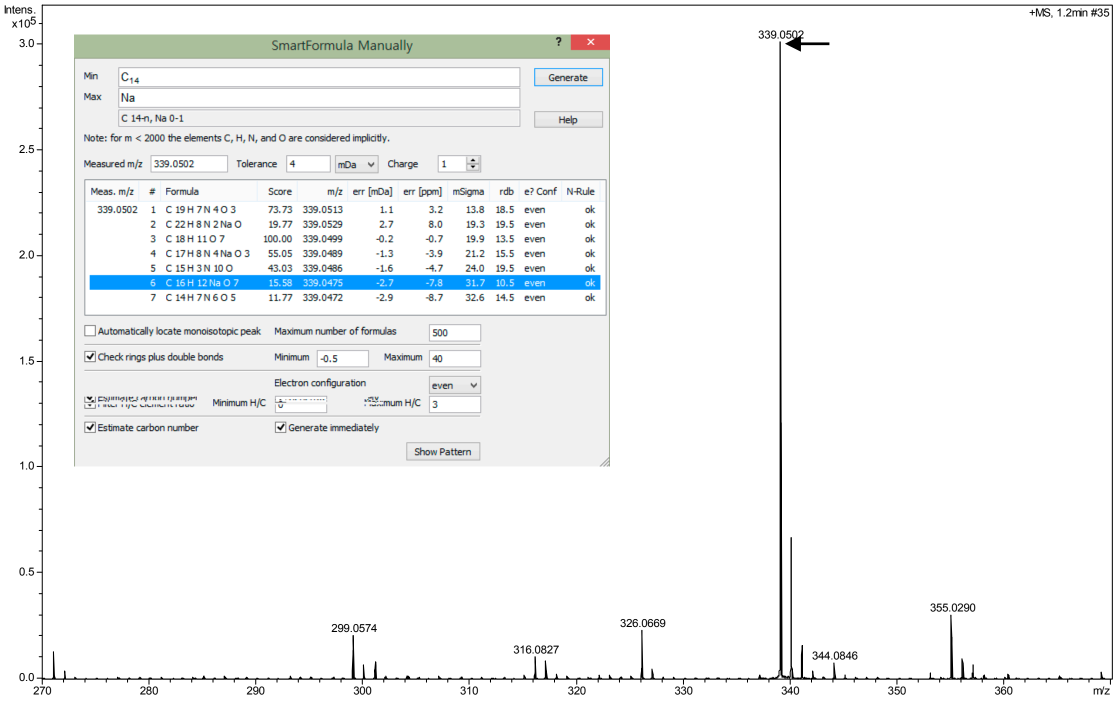


**Figure S5**. HR-ESI-Mass of compound **1**.
